# The siRNA Non-seed Region and Its Target Sequences Are Auxiliary Determinants of Off-Target Effects

**DOI:** 10.1371/journal.pcbi.1004656

**Published:** 2015-12-11

**Authors:** Piotr J. Kamola, Yuko Nakano, Tomoko Takahashi, Paul A. Wilson, Kumiko Ui-Tei

**Affiliations:** 1 Department of Surgery and Cancer, Imperial College London, London, United Kingdom; 2 Safety Assessment, GlaxoSmithKline R&D, Ware, Hertfordshire, United Kingdom; 3 Computational Biology, GlaxoSmithKline R&D, Stevenage, Hertfordshire, United Kingdom; 4 Centre for Radiation, Chemical and Environmental Hazards, Public Health England, Harwell Science and Innovation Campus, United Kingdom; 5 Department of Biological Sciences, University of Tokyo, Bunkyo, Tokyo, Japan; 6 Department of Computational Biology and Medical Sciences, University of Tokyo, Kashiwa, Chiba, Japan; Ottawa University, CANADA

## Abstract

RNA interference (RNAi) is a powerful tool for post-transcriptional gene silencing. However, the siRNA guide strand may bind unintended off-target transcripts via partial sequence complementarity by a mechanism closely mirroring micro RNA (miRNA) silencing. To better understand these off-target effects, we investigated the correlation between sequence features within various subsections of siRNA guide strands, and its corresponding target sequences, with off-target activities. Our results confirm previous reports that strength of base-pairing in the siRNA seed region is the primary factor determining the efficiency of off-target silencing. However, the degree of downregulation of off-target transcripts with shared seed sequence is not necessarily similar, suggesting that there are additional auxiliary factors that influence the silencing potential. Here, we demonstrate that both the melting temperature (Tm) in a subsection of siRNA non-seed region, and the GC contents of its corresponding target sequences, are negatively correlated with the efficiency of off-target effect. Analysis of experimentally validated miRNA targets demonstrated a similar trend, indicating a putative conserved mechanistic feature of seed region-dependent targeting mechanism. These observations may prove useful as parameters for off-target prediction algorithms and improve siRNA ‘specificity’ design rules.

## Author Summary

Small interfering RNAs (siRNAs) are double stranded RNA molecules designed to perfectly match the sequence of a target gene and silence its expression. The function is exerted through the RNA interference (RNAi) pathway and has revolutionised biological research due to its ease-of-use and high potency. While siRNAs were initially believed to be highly specific, they have subsequently been observed to interact with other, unintended messenger RNAs. However, the mechanistic details of this process remain poorly understood, and there is a paucity of strategies and guidelines directed toward mitigating this issue. To address this potential safety liability, we performed a comprehensive analysis of sequence characteristics of siRNA duplexes and their target regions. Results from luciferase-reporter assays and global expression data confirmed previous observations that the siRNA seed region is the primary determinant for off-target gene recognition and binding. Furthermore, our analysis revealed the important contribution of siRNA non-seed region, and its corresponding target sequences, to the potency of off-target knockdown. Similar results were observed in an equivalent evaluation of the miRNA-targeting mechanism, suggesting that the correlating features arise through an evolutionary conserved mechanistic factor.

## Introduction

**Fig 1:**
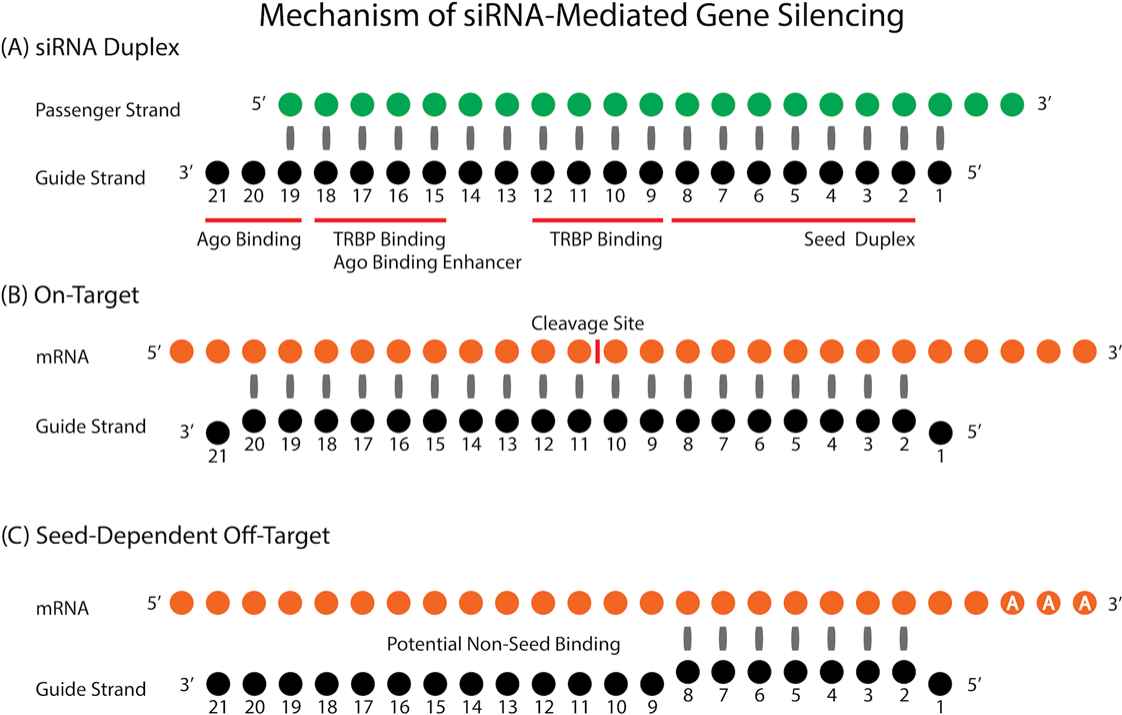
Graphical representation of siRNA molecule. **(A)** siRNA duplex with presumed binding sites for RISC proteins, Ago and TRBP [6]. **(B)** Interaction pattern between siRNA guide strand and on-target transcript and **(C)** interaction between siRNA guide strand and its seed-dependent off-target transcript. The mechanistic overview shows the guide strand of siRNA (A) as the strand which enters RISC, and acts in downstream silencing events (B and C). Nucleotide position numbers mirror those used in the text and consequent figures.

RNA interference (RNAi) is a highly regulated, evolutionarily conserved mechanism of post-transcriptional gene regulation. Small interfering RNAs (siRNAs), the intermediate utilised by this pathway, are 19 base pair (bp) long double stranded RNAs (dsRNAs), with 2 nucleotide (nt) 3’ overhangs (Fig 1A)[1]. When siRNAs are transfected into a cell, one of the siRNA strands (guide strand) is incorporated into the RNA-induced silencing complex (RISC) while the opposite strand (passenger strand) is degraded [2,3]. The activated siRNA-containing RISC (siRISC) recognises and binds to the target transcript in a sequence-specific manner (Fig 1B). The perfectly complementary region within the target transcript is then cleaved between the 10^th^ and 11^th^ nucleotide relative to the 5’ end of the guide strand [4]. This elegant, endogenous process has been extensively utilised in functional genomics studies and shows potential as a therapeutic platform [5].

When siRNAs were first shown to suppress gene expression in mammalian cells, the process was thought to be highly specific [1]. This belief was later challenged by reports of numerous sequence-specific off-target effects that could potentially induce a toxic phenotype [7,8]. The unintended targets were reported to share partial sequence complementarity with the guide strand and were found to primarily arise through a mechanism akin to miRNA targeting [9]. Specifically, 2^nd^ to 7^th^/8^th^ nt in the 5’ region (seed region) of siRNA recognises the unintended target gene within the 3’UTR of mRNA sequence (Fig 1C)[10]. While unintended interactions can also arise from the passenger strand entering RISC, in this study, ‘off-target effects’ will refer specifically to the guide strand seed-dependent type.

The combined false positive results in genomic studies and potential safety liabilities in clinical application are driving the need to design specific, off-target-free siRNAs. Strategies such as using multiple siRNAs with a shared-single target to dilute out siRNA-specific off-target activity or adjusting the concentration based on on-target potency both have their technical limitations [11,12], particularly in the case of therapeutics. Rational sequence design, with rules based on mechanistic insight, offers a more practical solution. The RISC is a ribonucleoprotein complex composed of multiple RNA-binding proteins, with Argonaute (Ago) playing a core role in the silencing process [13]. Ago interacts with the guide strand of an siRNA through two RNA-binding pockets in the PAZ (bound to 3’ end) and MID and PIWI (bound to 5’ end) domains [14]. We have found that the highly effective siRNA sequence simultaneously satisfy the following four rules; A/U at the 5’ end of the siRNA guide strand, G/C at the 5’ end of the passenger strand, AU richness in the 5’ one-third region of the guide strand, and the absence of long GC stretches [15]. Furthermore, the seed region is exposed on the Ago surface and is most likely mediating the initial target recognition through Watson-Crick base-pairing [16]. We also observed that the off-target efficiency is highly correlated with the thermodynamic stability of protein-free, seed-target duplex [15]. However, knockdown efficiency of seed-dependent off-targets with identical seed sequence but diverse non-seed region (positions 9–21) has shown varying levels of knockdown. While the seed region was confirmed as the primary driving force of off-target activity, our analysis also indicated a possible involvement of the non-seed region and its corresponding target sequences in the observed response [17].

To comprehensively estimate the contribution of sequence-based features to the potency of off-target effects, we correlated the thermodynamic profile (i.e. melting temperature [Tm]) of various subsections within siRNA guide strand with experimental results derived from off-target effect screens. Subsequently, we have utilized DNA microarray data generated using siRNAs that satisfy the sequence rules of highly effective siRNAs [15], to determine features associated with target sequence that influence the efficiency of off-target downregulation. Although little-to-no base-pairing is usually observed between siRNA non-seed region (nucleotides 9–20 in Fig 1C) and the corresponding off-target region, it was previously reported with miRNAs that such partial base-pairing is an additional factor that influences the efficiency of seed-dependent knockdown [18], and was thus included in our analysis. To assess the general applicability of our findings for miRNA silencing, a dataset of experimentally verified miRNA targets was also studied to determine potential conservation of observed features. Understanding and utilization of parameters of thermodynamic control, along with target recognition features, will provide a reliable and practical method to design effective and safe siRNAs, for both research and therapeutic applications.

## Results

### Hybridization thermodynamics of three sub-regions in the siRNA duplex correlate with potency of off-target effect

**Fig 2:**
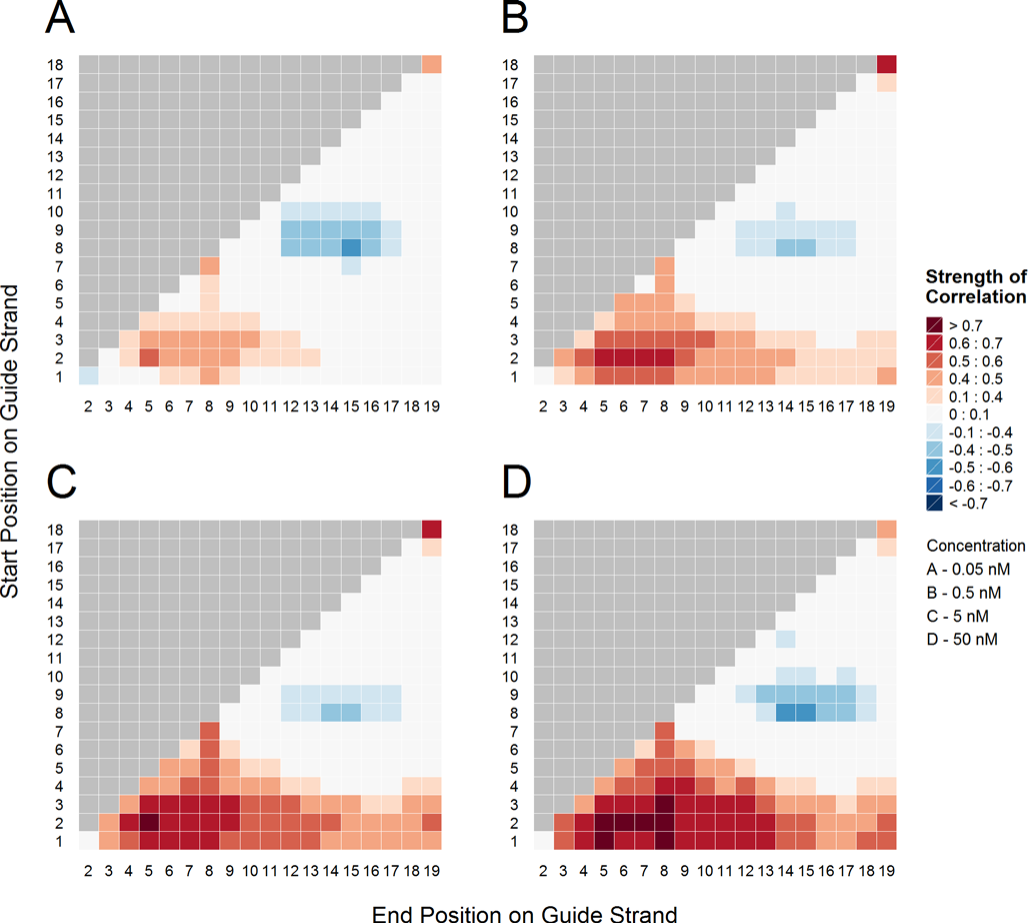
Correlation between *T*_m_ values of sequence subsections within siRNA duplexes and the corresponding off-target silencing efficiency. The start of the subsection within a duplex is plotted on the Y axis (‘Start Position’) whereas the end of the subsection is plotted on the X axis (‘End Position’). The position numbering mirrors that used in Fig 1. The analysis was performed separately for each siRNA concentration–**(A)** 0.05, **(B)** 0.5, **(C)** 5 and **(D)** 50 nM. The siRNA sequences used in the analysis, together with corresponding knockdown percentages, are listed in S1 Table.

To investigate the contribution of hybridization thermodynamics to the efficiency of RNA interference in detail, the correlation between melting temperature (Tm) of all possible sequence subsections within siRNA duplex and corresponding off-target knockdown measured by luciferase-reporter assays was plotted as two dimensional arrays (i.e. heatmap). Melting temperature has been shown to be a strong predictor of thermodynamic stability of seed-target duplex [17,19] and was the measure of choice in this study. The approach allowed us to distinguish three, well separated clusters which sequence composition correlated with RNAi effects (Fig 2): [1] positively correlated cluster in the siRNA seed region (nucleotides 2–8), [2] negatively correlated cluster in the non-seed region (specifically nucleotides 8–15), [3] positively correlated 3’ termini position (nucleotides 18–19). The correlation coefficient (r) of the ‘seed’ cluster exceeded 0.7 in results from higher siRNA concentrations (Fig 2C and 2D). The high degree of overlap between plots at different concentrations of siRNAs increases confidence that the observations are significant, and the results confirmed our previous report, which demonstrated strong positive correlation between *T*_m_ values of seed-target duplex and off-target silencing efficiency. Longer sequence subsections that overlap a highly correlated shorter region, are more likely to show higher correlation. It is thus appropriate to concentrate on the subsections showing the highest correlation coefficient and the shortest length. Interestingly, nucleotides 2–5 and not the whole seed (nucleotides 2–8) showed the highest positive correlation at all siRNA concentrations (from r = 0.51 at 0.05 nM to r = 0.78 at 50 nM, all p-values < 0.01), suggesting that positions 2–5 may be the most crucial in the interaction between the guide strand and off-target transcripts. The correlation between 3’ termini positions and knockdown efficiencies exceeded r = 0.7 in results from assays at 0.5 and 5 nM concentrations. Asymmetry in thermodynamics of 3’ and 5’ termini bases in the siRNA duplex determines which strand will be preferential selected and loaded into RISC [17,20,21]. The 3’ terminal nucleotide is known to be anchored in the PAZ domain of the Ago protein [22]. However, it might be found that the 3’ terminal region is more strongly associated with RISC incorporation compared to 5’ terminal region. While the contribution of seed region binding and 3’ terminal base to silencing potency has been well established [15,17], our results revealed a novel negatively correlated cluster within the non-seed region of siRNA. The correlation between *T*_m_ values at non-seed nucleotides 8–15 and off-target knockdown efficiencies was relatively lower but evident at all tested concentrations (r = ~-0.5, all p-values < 0.01).

### GC content in the target sequence corresponding to siRNA non-seed region affects off-target efficiency

**Fig 3:**
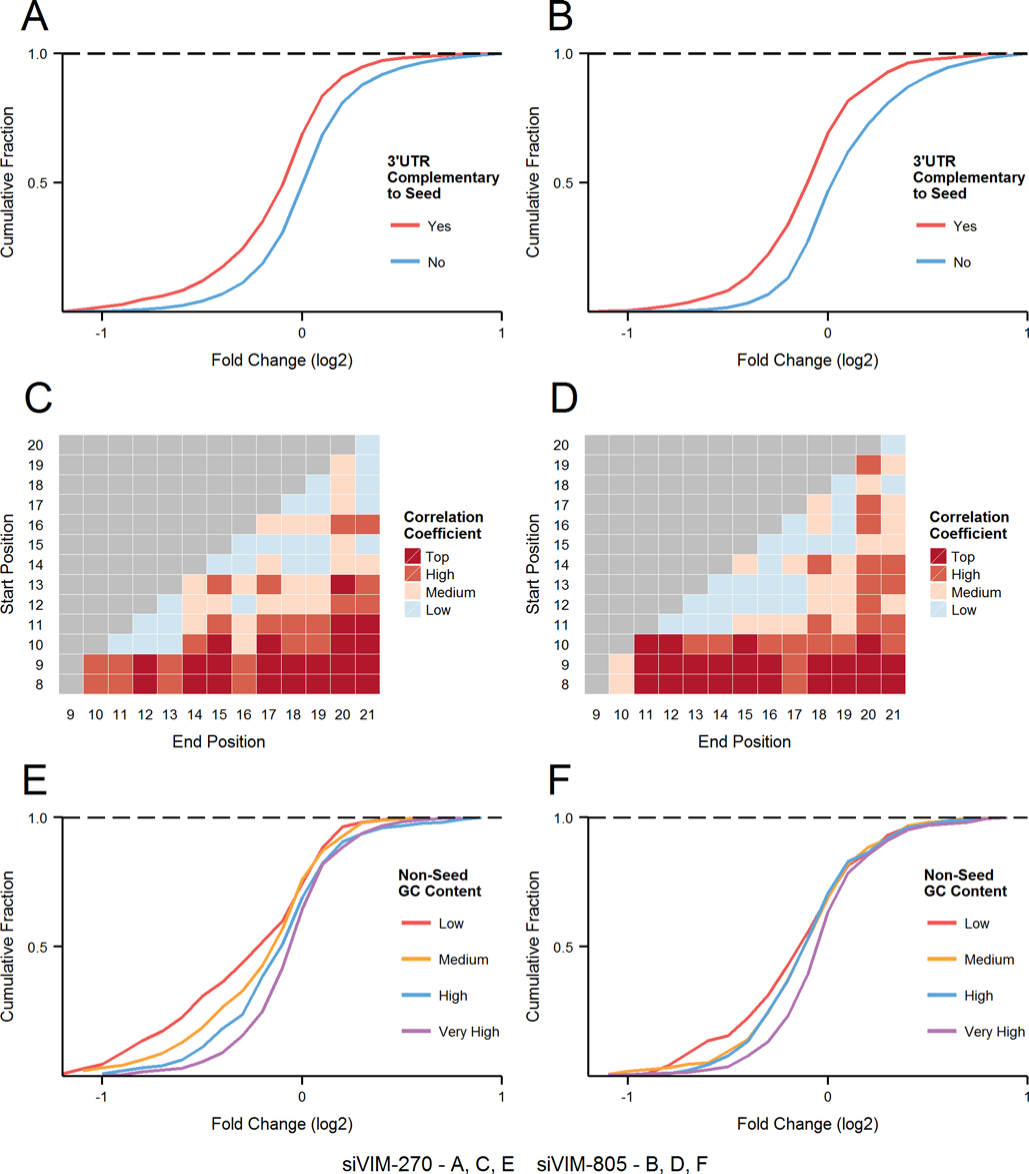
Seed and non-seed region-dependent off-target effect analyses for siVIM-270 and siVIM-805. **(A, B)** Expression profiles of off-target genes with 3’UTR sequences that perfectly match the corresponding siRNA seed region (i.e. off-targets) were compared to genes without such sequence. **(C, D)** The correlation between GC content in all the subsections within mRNA targets of siRNA non-seed region (8–21) and fold change of off-target effect was calculated in a similar manner to that shown in Fig 2. The correlation coefficient was grouped using quantiles as boundary values and target position corresponds to the numbering shown in Fig 1. GC contents in the non-seed regions at positions 8–15 showed the highest correlation with off-target effect (Fig 3C; siVIM-270, r = 0.22, p-value = 1.13E-12, Fig 3D; siVIM-805, r = 0.15, p-value = 1.57E-05). **(E, F)** Off-target transcripts for siVIM-270 and siVIM-805 were divided into four groups defined by the number of GC nucleotides in their non-seed regions (positions 8–15). Quantiles were used as the boundary values for classification; ‘Low’ (GC content < 3), ‘Medium’ (GC content = 3), ‘High’ (GC content = 4) and ‘Very High’ (GC content ≥ 5)(out of a total of 8). The number of off-target genes was 1065 for siVIM-270 (E) and 823 for siVIM-805 (F).

While the thermodynamics analysis of siRNA duplex revealed significant contribution of non-seed sub-region to off-target effect, a question remained regarding the potential effect of off-target sequence (especially at the off-target sub-section contributing to the observed negative correlation in Fig 2). Although the interaction between guide strand and off-target transcripts is mainly driven by base-pairing at the seed region, there is a likelihood of limited interaction between sequences at the siRNA non-seed region (Fig 1C). To comprehensively test this possibility, we analysed global gene expression changes induced by two siRNAs, siVIM-270 and siVIM-805 designed against human Vimentin (VIM) gene, which were previously shown to have potent off-target effects [17]. Putative off-targets were predicted based on perfect complementarity between the siRNA seed (nucleotides 2–8) and 3’UTR sequences derived from the longest transcript of every protein-coding gene. While perfect complementarity between seed and target is not always required for inducing off-target effect, analysis of off-target effects with varied seed binding (and thus different binding energy) might introduce additional variability, and further complicate the analysis. First, expression profiles of genes annotated as the putative off-targets were compared to non-off-target genes without seed-matched regions. For both siRNAs, there is a clear separation between the two groups of transcripts, with seed-matched sequences showing significantly lower expression profile relative to the remaining genes without seed-matched sequences (Fig 3A; siVIM-270, KS-test d-value = 0.21 with p-value < 2.2E-16, Fig 3B; siVIM-805, KS-test d-value = 0.24 with p-value < 2.2E-16).

The level of downregulation was different among off-target transcripts, which may be partly explained by the variation in the sequence of their non-seed region. While potential and limited base-pairing can be found between siRNA non-seed region and its corresponding target sequences, the currently established calculation procedure of thermodynamic profiles is not applicable for such discontinuous duplexes. The correlation profiles between all possible subsections within target sites corresponding to siRNA non-seed region and off-target effects were thus created based on sequence characteristics measured through GC content (Fig 3C and 3D). While all off-target genes share the same sequence at positions 2–8, which is complementary to siRNA seed region, the analysis included nucleotide at position 8 as it was found to be important in analysis shown in Fig 2. The correlation based on luciferase-reporter assay (Fig 2) was calculated using % of repression whereas the results based on DNA microarrays (Fig 3) were calculated using fold change. Lower fold change translates into greater downregulation and thus the correlations in the two analyses are opposite in direction. With the difference between values being much smaller compared to the data shown in Fig 2, we have ranked and classified them into ‘Low’, ‘Medium‘, ‘High’ and ‘Top’ groups using quantiles. The degree (i.e. strength) of correlation in both plots is strongest around the bottom region, indicating that sequence subsections encompassing most of the non-seed region show the strongest positive relationship with fold change (Fig 3C and 3D). The patterns observed in global expression data derived from samples treated with siVIM-270 and siVIM-805 confirmed the results from luciferase-reporter assays–siRNA with high GC content in the non-seed region have ‘weaker’ off-target effects. While the top correlations are not as high as those observed with the seed region (Fig 2), they are both statistically and biologically significant (siVIM-270, r = 0.23, p-value = 1.2E-13; siVIM-805, r = 0.17, p-value = 9.3E-07). Comparing results from both siRNAs revealed that the common positions with top correlation were 8–15 (p-values < 0.01) followed by 9–15 (p-values < 0.01). The former was also identified as the optimal region in the luciferase-reporter assay (Fig 2) and was thus selected as the sequence subsection for all further analyses. The GC content of positions 8–15 was then used to divide off-target transcripts into four groups (based on quantiles), whose expression patterns were plotted as cumulative distributions (Fig 3E and 3F). From left to right, a clear pattern can be seen where targets with lower GC content in the subsection corresponding to siRNA non-seed positions 8–15, show greater downregulation. For each siRNA, the Kolmogorov-Smirnov test was performed between the ‘Low’ and ‘Very High’ groups, and generated statistically significant results for both siRNAs (Fig 3E; siVIM-270, KS-test d-value = 0.29 with p-value of 7.9E-07, Fig 3F; siVIM-805, KS-test d-value = 0.2 of p-value = 0.003). The higher number of predicted off-target transcripts for siVIM-270 (particularly for off-target transcripts with 1 or 2 GC non-seed matches) most likely determines the corresponding distinct profile.

### Non-canonical interaction between guide strand non-seed region and the corresponding target might influence off-target effects

**Fig 4:**
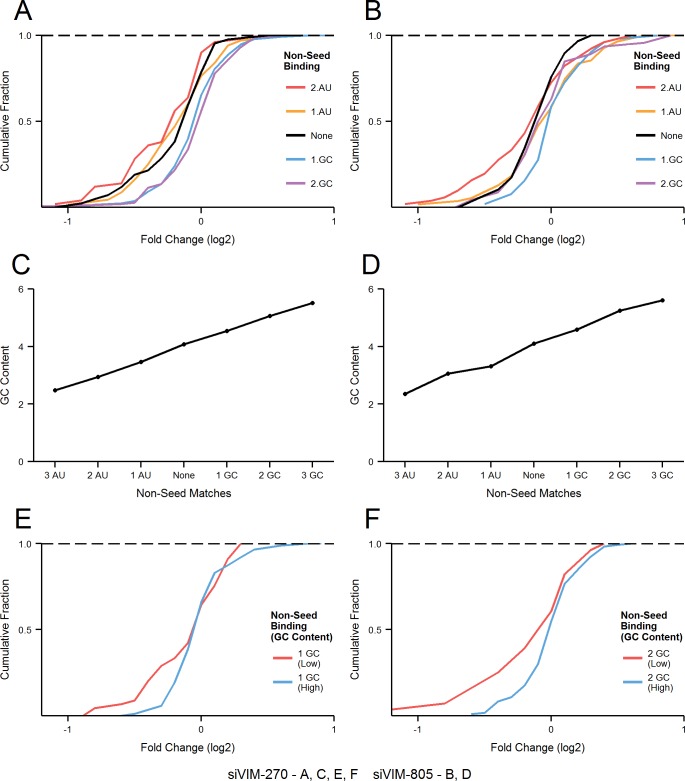
Analysis of off-target effects based on sequence similarity between an siRNA non-seed region and its corresponding target sequences. **(A, B)** Cumulative distribution of off-target transcripts grouped by their non-seed base-pairing (Fig 4A; siVIM-270 off-target effects, 2AU (70), 1AU (52), None (27), 1GC (133), 2GC (151), Fig 4B; siVIM-805 off-target effects, 2AU (55), 1AU (52), None (20), 1GC (52), 2GC (46)). Off-target transcripts with more than 2AU or 2GC match were omitted due to their low number. **(C, D)** The average GC contents for non-seed region (positions 8–15) were calculated for each group of off-target transcripts. **(E, F)** The cumulative distribution of off-target transcripts of siVIM-270 with 1GC match (133 transcripts)(E) and 2GC matches (151 transcripts)(F), were sub-divided based on their GC contents at positions 8–15. ‘Low’ subgroups have GC content lower than the average while ‘High’ subgroups have GC content higher than the average.

As the off-target effects were identified based on perfect complementarity to the seed region (positions 2–8) and both terminal nucleotides of siRNA are known to be incorporated into Ago protein (positions 1 and 21)[22], the potential base-pairing between siRNA and off-target mRNA was determined via positions 9–20 (i.e. 3’ region). The number of GC and AU base-pairs was calculated for each off-target sites, which allowed the transcript to be placed into five distinctive groups; 2AU, 1AU, None (i.e. no found matches), 1GC and 2GC. Although most of these groups were relatively small, the distribution of each group for both siVIM-270 (Fig 4A) and siVIM-805 (Fig 4B) showed a clear separation and comparable ordering. The higher numbers of AU matches resulted in greater downregulation while the higher numbers of GC matches showed lower downregulation. The profile of off-target effects without possible non-seed binding was located between the AU and GC groups. To better understand this pattern, average GC content of positions 8–15 was calculated for each group of transcripts, and plotted in the same order as observed in Fig 4A and 4B. A very strong relationship was observed between the average GC contents of sequences corresponding to siRNA non-seed positions 8–15 and the off-target transcripts containing a particular non-seed binding pattern. Off-target transcript with AU matches in region 9–20 have on average lower GC content in region corresponding to the siRNA positions 8–15, while those off-target transcript with GC matches have on average higher GC content. The ordering based on average GC values of the groups shown in Fig 4C and 4D closely mirrors that in Fig 4A and 4B.

To further explore the relationship between off-target effects and GC content in the target region corresponding to the siRNA positions 8–15, we investigated the cumulative distribution of off-target subgroups according to their GC content. The average GC content for each of the group was used to further divide the transcripts with a particular non-seed binding pattern into two subgroups: one with GC contents less than the mean (‘Low’) and the other with GC contents more than the mean (‘High’). Unfortunately, in majority of cases, the subgroups were too small to perform an accurate analysis. We have thus limited our analysis to the two largest groups (Fig 4E; siVIM-270, 1GC match, 133 off-target transcripts, Fig 4F; siVIM-270, 2GC matches, 151 off-target transcripts). In both groups, the results clearly indicated that off-targets with lower GC content in the region corresponding to siRNA positions 8–15 show a greater downregulation. It is difficult to verify whether base-pairing in the siRNA non-seed region occurs in a ‘justified’/canonical (e.g. binding occurs between nucleotides directly opposite each other, as calculated in Fig 4A and 4B) or ‘slided’/non-canonical (e.g. binging occurs between nucleotides not opposite each other, which could explain the pattern observed in all plots in Fig 4) fashion. Nevertheless, it was apparent that the high GC contents in the off-target sequences corresponding to siRNA positions 8–15 reduces the potency of off-target effects. Based on the ordering of the groups in Fig 4A–4D it seems as the ‘justified’ base-pairing does not fully occur/affect the seed dependent interaction. Rather, the groups indirectly represent the GC content of the target region as regions with high GC content are more likely to have more GC base-pairs with siRNA non-seed region (as shown in Fig 4C and 4D).

### GC content of target sequence corresponding to miRNA non-seed region may influence silencing potential

Since our present observations demonstrate that siRNA non-seed region may be a novel auxiliary determinant of siRNA-based off-target effect, we extended our investigation to miRNA-based silencing events. While the availability of accurate estimates of the silencing potential of a particular miRNA is severely limited due to their endogenous origin, our analysis took advantage of a database that summarizes their experimentally confirmed targets [23]. The presence or absence of a target in the list formed the basis of our comparison. Twenty five miRNAs with the highest number of experimentally-ascertained targets, including those with perfect and non-perfect seed-complementarity, were selected for the analysis. Putative miRNA targets were predicted as for siRNA (i.e. perfect complementarity between 3’UTR sequence and seed region), and consequently intersected with the list of confirmed hits (miRNAs with at least 500 predicted targets were considered). Predicted off-target genes that were experimentally confirmed were placed in ‘Validated Targets’ group while predicted off-target genes that were not experimentally validated were placed in ‘Remaining Genes’ group. Average GC content of the region corresponding to the miRNA positions 8–15 for all genes was calculated separately for each group and compared (Table 1). Of the 25 evaluated miRNAs, 21 had lower GC content (nucleotides 8–15) in the ‘Validated Targets’ group compared to the ‘Remaining Genes’ group. While a more thorough approach is needed to accurately explore the relationship (e.g. target prediction not limited to those with perfect seed complementarity, analysis based on correlation between GC content and quantified silencing potential, comparison between expression profiles), the results provide evidence that the phenomenon observed with siRNA might represent a mechanistic feature which also regulate the efficiency in miRNA target silencing.

**Table 1:** Contribution of miRNA non-seed region to gene silencing. The list of putative targets perfectly matching the miRNA seed region (positions 2–8) was intersected with a list of experimentally validated miRNA targets [23]. Genes present on both lists were placed in the ‘Validated Targets’ group, while genes predicted to interact with miRNA but which have not been experimentally confirmed, were placed in ‘Remaining Genes’ group. The GC content in the positions 8–15 was calculated for both groups and compared. The difference was calculated by subtracting values of the ‘remaining’ group from the values in the ‘validated’ group.

miRNA ID	Length	Validated Targets (GC)	Remaining Genes (GC)	Difference
hsa-miR-24-3p	22	3.77	4.49	-0.71
hsa-miR-93-5p	23	3.08	3.57	-0.5
hsa-miR-16-5p	22	2.61	3.08	-0.46
hsa-miR-98-5p	22	3.60	4.00	-0.39
hsa-miR-7-5p	23	3.51	3.90	-0.39
hsa-miR-17-5p	23	3.19	3.56	-0.37
hsa-miR-34a-5p	22	4.06	4.41	-0.35
hsa-let-7b-5p	22	3.66	4.00	-0.34
hsa-miR-192-5p	21	2.23	2.56	-0.33
hsa-miR-215-5p	21	2.22	2.54	-0.32
hsa-miR-744-5p	22	4.90	5.22	-0.32
hsa-miR-124-3p	20	3.44	3.74	-0.29
hsa-miR-21-5p	22	2.06	2.33	-0.27
hsa-miR-193b-3p	22	3.79	4.05	-0.26
hsa-let-7a-5p	22	3.72	3.98	-0.26
hsa-miR-149-5p	23	4.10	4.32	-0.22
hsa-miR-331-3p	21	4.46	4.67	-0.21
hsa-miR-155-5p	23	2.16	2.31	-0.15
hsa-miR-92a-3p	22	3.56	3.66	-0.1
hsa-miR-30a-5p	22	2.25	2.35	-0.09
hsa-miR-26b-5p	21	2.27	2.33	-0.05
hsa-miR-186-5p	22	2.40	2.37	0.03
hsa-miR-877-3p	21	4.12	4.07	0.05
hsa-miR-222-3p	21	2.54	2.41	0.13
hsa-miR-335-5p	23	4.01	3.79	0.22
	Average	3.27	3.51	-0.24

## Discussion

To uncover the rational design rules of potent siRNAs, several studies have attempted to reveal sequence-dependent features within the siRNA molecule that correlate with efficiency of RNAi [21,24]. To better understand the regulatory mechanisms of off-target effects we exhaustively analysed the contribution of each subregion within the siRNA duplex to clarify their relative contributions to potency of off-target silencing. The significance of seed region (nucleotides 2–8) thermodynamic stability in the process of off-target effects has been thoroughly explored [17]. Our detailed study revealed that the siRNA seed region encompassing nucleotides 2–5 has the highest positive correlation with off-target effect (Fig 2). This result may not be so surprising, since the crystal structure of human Ago2 revealed that nucleotides 2–6 of the guide RNA are splayed out and stably positioned on the MID and PIWI domains in an A-form conformation for base pairing with target mRNAs [25]. This preorganization into A-form like geometry significantly increases affinity for the target RNA [26,27]. However, there is a kink between nucleotide 6 and 7 that breaks the A-form structure. These types of conformational change are considered to be related to the importance of base-pairing of nucleotide 7 with off-target transcripts. Furthermore, base-pairing at nucleotide 8 can subsequently stabilise the duplex, although mismatch at this position does not perturb the duplex formation at positions 2–7. A more recent study has also revealed that initial target scan performed by Ago2 identifies target sequences complementary to nucleotides 2–4 of the miRNA [28]. Thus, the efficiencies of off-target effects might show a stronger correlation with the thermodynamic stabilities of nucleotides 2–5 compared to those of nucleotides 2–8.

The majority of siRNAs used in this study have A/Us at 5’ ends of the guide strands (positions 1–2) and G/Cs at the 5’ end of the passenger strands (positions 18–19)(see S1 Table). The calculated *T*_m_ values at nucleotides 1–2 showed no or little correlation with the efficiencies of off-target effects, while those at nucleotides 18–19 showed strong positive correlation (Fig 2). One of the most widely accepted design algorithms of functional siRNAs for on-target repression is based on the asymmetrical thermodynamics of 5’ end of the guide strand (position 1) and 5’ end of passenger strand (position 19). The strand with lower base-pairing stability at its 5’ end is preferentially incorporated and retained in the RISC [17,20,21]. Selective entry of the intended guide RNA strand into RISC significantly increases the efficiency of target gene cleavage, while entry of the opposite passenger strand is undesirable. Furthermore, A/U at the guide strand 5’ terminal shows the 30-fold higher affinity for anchoring in the Ago pocket compared to G/C [29]. While it is possible that 5’ terminal A/U of the guide strand is important for anchoring in the Ago pocket, the thermodynamic profile of nucleotides 1–2 may have little to no correlation with off-target efficiencies. Such an observation is also consistent with previous experiments, indicating no contribution of A/U base pairs at position 1 to the off-target efficiency [9,15]. In contrast, the calculated *T*_m_ values at nucleotides 18–19 showed a high correlation with off-target silencing efficiencies across all siRNA concentrations (from r = 0.45 to r = 0.64, all p-values < 0.01). The results indicate that asymmetrical thermodynamics of both ends of siRNA duplex is predominantly regulated by the 3’ terminal nucleotide rather than the 5’ terminal nucleotide. Alternative explanation can be based on structural analysis, which showed relatively stronger electron density, indicating stronger binding, in the 5’ pocket of the PIWI domain with G/C nucleotide [29] relative to 3’ binding pocket of the PAZ domain [25].

The most striking result of this study was that the siRNA non-seed region is also significantly involved in determining the efficiency of off-target effect. The negative cluster in siRNA analysis (Fig 2) overlapped sequences positioned between nucleotides 8 and 17, which agrees with structural studies showing that nucleotides 18–21 are held away from the target RNA [30,31]. The base-pairing stability in the non-seed region positioned at 8–15 showed the highest negative correlation with off-target activity (r = ~0.5 across all concentrations, all p-values < 0.01) in duplicated experiments using two different siRNAs, siVIM-270 and siVIM-805. This tendency of correlation is apparent even at the lowest concentration of 0.05 nM (Fig 2A). The non-seed region did not, however, show an increase in correlation coefficient with increasing siRNA concentration. In contrast, the effect of the seed region was dose-dependent, suggesting that the initial interaction through seed-region is a rate-limiting step, with consequent interactions being of lesser hindrance. Interestingly, GC content in target sequence corresponding to siRNA non-seed region (nucleotides 8–15) showed the highest correlation with off-target efficiencies (Fig 3), which appears to be independent of a ‘justified’ base-pairing within the non-seed region (nucleotides 9–20)(Fig 4). Nevertheless, results from both analyses showed non-seed region 8–15 as the highest negatively correlating feature, which suggest that interaction between those regions might be behind the effect. It is thus likely that the regions interact via base-pairing with the nucleotides not exactly opposite each other. Such event might disrupt the seed-target duplex by pulling apart both strands. The further away from the seed region, the less effect any potential base pairing may have on the stability of the seed duplex. Alternatively, high GC contents of both regions might interfere with initial recognition (e.g. binding competition between seed and non-seed), affect protein binding (with RISC or other as yet uncharacterised molecule) or alter downstream events, which translate into reduced OTE knockdown.

A recent comprehensive study of miRNA target interactions reported widespread non-canonical interaction between miRNA non-seed region and its target mRNA sequences [32]. Similar to siRNA, miRNA identifies and binds to its targets primarily through its seed region. Our analysis based on dataset of experimentally validated miRNA targets [23] indicate that validated targets have on average lower GC content in the sequence corresponding to miRNA non-seed region (nucleotides 8–15) relative to predicted off-target effects that were not experimentally validated (Table 1). Given these combined observations we propose that the non-seed region may function through a conserved mechanism shared by both miRNA and siRNA molecules.

**Fig 5:**
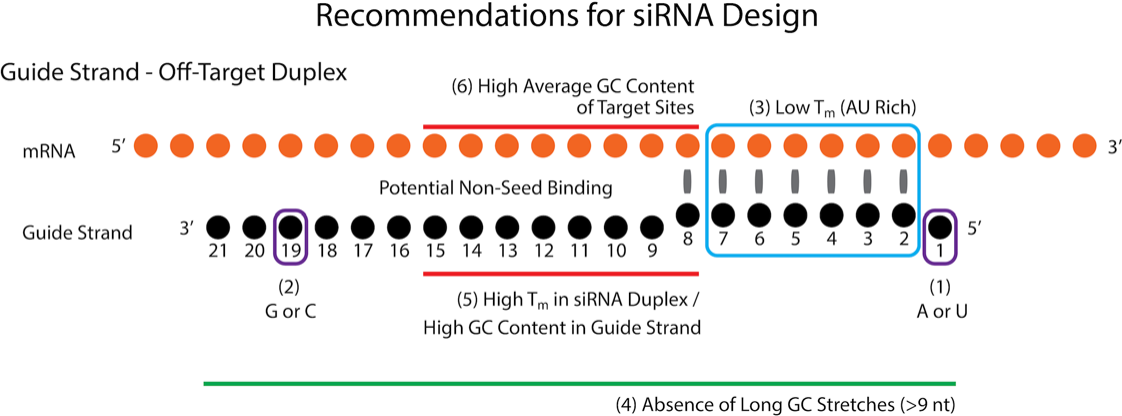
Summary of ‘highly effective’ and ‘off-target effect-reduced’ design rules for siRNA molecules. Based on our previous analysis and the results shown in Figs 2–4, we recommend designing molecules with: (1) A or U at position 1, (2) G or C at position 19, (3) low predicted *T*_m_/AU richness at positions 2–7, (4) absence of GC stretches longer than 9 nt, (5) high predicted *T*_m_ in siRNA duplex/high GC content in guide strand at positions 8–15, and (6) high average GC content for all off-target sequences corresponding to positions 8–15 in the siRNA non-seed region.

We have previously reported that highly functional siRNAs satisfy the following four rules [15]: (1) A or U at 5’ end of siRNA guide strand, (2) G or C at 5’ end of siRNA passenger strand, (3) AU richness at 5’ one-third region of siRNA guide strand, (4) absence of any GC stretches more than 9 nt in length. Based on our current and previous analyses [17], we recommend that additional emphasis is placed on three design aspects tailored specifically towards minimising off-target effects (summarised in Fig 5): (3’) low *T*_m_ in the siRNA seed region (nucleotides 2–5 to 2–7), (5) high *T*_m_ in siRNA duplex/high GC content in the guide strand within non-seed region (nucleotides 8–15), (6) high average GC content for target sequences corresponding to nucleotides 8–15 of the siRNA guide strand. Although the first ‘specificity’ rule (3’) overlaps with the third functional siRNA rule (3) the revised recommendation is more precise regarding the location of AU base-pairs (nucleotides 2–5 to 2–7). While the first two specificity indications, (3’) and (5), will be easily satisfied by calculating *T*_m_ values in the selection step of a particular siRNA duplex, the third (6) is inevitably varied and will require a rigorous off-target effects analysis to be performed to select the most promising designs. This can be accomplished by extracting the sequences upstream of the sites predicted as targets for siRNA seed region and calculating their average GC content. Data presented in Figs 2–4 indicate that adhering to these rules will reduce global off-target effect liability.

RNAi is a cascade of protein-RNA interaction events, with reaction rate and efficiency of each step directly affecting the downstream event. The potency of siRNA-mediated knockdown is largely dependent on siRNA sequence, with particular features within the molecule affecting the rate of reaction steps [24]. This phenomenon has led to the general acceptance of several ‘design rules’ of functional siRNA [33], which may reflect a mechanistic feature of the RNAi. While high on-target knockdown is essential, it is important to address the problem of unintended off-target effects [34–36]. Previously reported factors which contribute to the potency of off- (strength of base-pairing between seed region and target) and on-target (asymmetry in end stability of siRNA duplex) functions [15] were confirmed in this study. Additionally, *T*_m_ in the siRNA duplex at non-seed positions 8–15 and the GC content of target sequence corresponding to the siRNA non-seed positions 8–15 were found to be correlated with the off-target downregulation. The results were also replicated in a database of confirmed miRNA targets suggesting that the contribution of the non-seed region to silencing event arise through a conserved mechanistic feature of seed-dependent targeting mechanism.

## Methods

### Oligonucleotide sequence

Data from our previously reported off-target effect assays of 32 siRNAs was pooled, averaging values for duplicate measurements, setting the maximum luciferase response at 100% and transforming into knockdown % to facilitate interpretation [17,19]. The measurements are based on reporter plasmids expressing *Renilla* luciferase gene with three tandem repeats inserted into the 3’UTR region of the mRNA. The repeat sequence contains a region of perfect complementarity to the seed region of corresponding siRNA (positions 2–8 in Fig 1C), while the remaining regions are intentionally devoid of homology to the non-seed region. The sequence details of all siRNAs, together with corresponding % knockdown at each concentration, are listed in S1 Table.

### Thermodynamics analysis

Thermodynamic profiles were created by evaluating every subsection within the guide strand of siRNA (sequences are listed in S1 Table). For every subsection a *T*_m_ was calculated using a nearest neighbour model with thermodynamics parameters for RNA-RNA interaction based on Xia et al. [37]. As the comparison is performed for ‘internal’ sequence, the helix initiation factor and symmetry correction were both omitted. The final *T*_m_ equation used was:

$$T_{m}({}^{\circ}C)=\frac{1000\;x\;\Delta H}{\Delta S+In\frac{Ct}{4}}-273.15+16.6\;\log[{Na}^{+}]$$

Where ΔH is the sum of enthalpy changes (kcal mol^-1^), ΔS is the sum of entropy changes (kcal mol^-1^ K^-1^), Ct is the total molecular concentration of the siRNA (variable) and [Na^+^] is the sodium ion concentration (100 mM).

### Microarray data analysis

The microarray analysis was performed according to our previous report [17]. Briefly, HeLa cells were transfected with either of 50 nM siVIM-270 or siVIM-805. Total RNA was purified and hybridized to Human Genome U133 Plus 2.0 GeneChip (Affymetrix). RNA from mock-transfected cells was used as a control. The transcript expression value was calculated using Microarray Suite 5.0 (MAS5)[38] with quantile normalization [39,40]. The 3’UTR sequence used in off-target transcript prediction and analysis were generated based on gene sequences and coordinate, and probe mapping annotation available in the Ensembl repository [41]. Several steps were taken to ensure accuracy and non-redundancy of the downstream analysis: probes overlapping multiple genes were removed, probes annotated as ‘absent’ or ‘marginal’ by MAS5 analysis suite were omitted, a single probe was selected per gene when several were available, a single 3’UTR sequence was selected per gene (based on the longest transcript) in case of multiple splice variants with annotated UTR regions. To reduce potential variability, a gene was treated as a target only when its 3’UTR sequence had a fragment perfectly matching the seed region of the siRNA or miRNA molecule (positions 2–8). Genes with more than one putative prediction were also excluded from further analysis to ensure independence of analysed samples. A custom script was prepared to locate sites within 3’UTR sequences with perfect match to siRNA seed region and extract the flanking sequences for further analyses. Potential base-pairing in the non-seed region was calculated based on region 9–20 as the nucleotide in position 21 is hidden in the PAZ domain of the Ago protein [22]. GC content was calculated for all possible subsections within predicted targets corresponding to siRNA guide strand non-seed region (positions 8–21)(Fig 1C), mirroring the analysis method of the guide strand described in the ‘Thermodynamics Analysis’. Results for particular sets of off-target effects were plotted as empirical cumulative distribution functions, which is a convenient method of visualizing differences in distribution and expression patterns between analysed groups. Kolmogorov-Smirnov test (KS-test) was used to quantify the separation between plotted cumulative distributions and test its significance.

### Correlation and visualization

The strength of a linear association between melting temperatures of all possible subsections within siRNA duplexes and their off-target efficiencies measured by *Renilla* luciferase assay was calculated separately for all siRNA concentration (0.05, 0.5, 5 and 50 nM). Similarly, the correlation between GC contents of all possible target sequences corresponding to the various siRNA non-seed subsections, and the fold changes derived from DNA microarray experiments, was calculated for siVIM-270 and siVIM-805. The profiles were plotted as heatmaps, as a convenient method to represent and interpret two dimensional data (Figs 2 and 3C and 3D). The nucleotide position numbering is identical to that used in Fig 1 (Y axis–start position on the guide strand/target, X axis–end position on the guide strand/target). Two different correlation tests were used: Pearson product-moment correlation and Spearman rank correlation coefficient. Though the results from both methods were largely overlapping, the latter was chosen for final plotting as it generated more consistent and defined clusters, most likely due to the monotonic nature of the data. All statistical calculations and plotting were performed in R.

## Supporting Information

S1 Table A list of siRNA molecules used in the heatmap analysis. (DOCX)
