# Supplementary material for: The siRNA Non-seed Region and Its Target Sequences Are Auxiliary Determinants of Off-Target Effects
Source: PLoS Comput Biol. 2015 Dec 11;11(12):e1004656. doi: 10.1371/journal.pcbi.1004656 (PMC4676691; doi:10.1371/journal.pcbi.1004656)
Supplement: S1 Table — (DOCX) [file pcbi.1004656.s001.docx]

| Sequence Detail | | % Knockdown | | | |
| --- | --- | --- | --- | --- | --- |
| siRNA ID | Guide Strand (5’ -> 3’) | 0.05 nM | 0.5 nM | 5 nM | 50 nM |
| siTUBA2-714 | UCAAAUCGCAGGGAGGCCGUG | 0 | 0 | 19.5 | 38.9 |
| siLuc-1048 | AUCCCCCUCGGGUGUAAUCAG | 0 | 16 | 64 | 70 |
| siVIM-596 | AAGAUUGCAGGGUGUUUUCGG | 12 | 27 | 41 | 51 |
| siOct-821 | AUCUUUUGCCCUUCUGGCGCC | 6 | 2 | 26 | 35 |
| siVIM-1261 | AUUAGUUUCCCUCAGGUUCAG | 0 | 12 | 27 | 26 |
| siITGA10-2803 | UAUUGGAUGUAGGCUGAGGUC | 7 | 37.7 | 33.5 | 14.8 |
| siCLTC-4819 | UAAUUUAUCCACCUUUGUCAA | 0 | 9 | 8 | 7 |
| siGRK4-934 | AAUAUUCUCAGGCUUCAAGUC | 0 | 0 | 0 | 0 |
| siFL-774 | AUUAAGACGACUCGAAAUCCA | 1 | 4 | 25 | 24.5 |
| siLuc-36 | UCUUCCAGCGGAUAGAAUGGC | 2 | 10 | 19 | 51 |
| siFL2-153 | UCGAAGUAUUCCGCGUACGUG | 0 | 17 | 33 | 45.5 |
| siVIM-270 | UUGAACUCGGUGUUGAUGGCG | 3 | 46 | 83 | 75 |
| siVIM-1128 | UAUUCACGAAGGUGACGAGCC | 0 | 0 | 4.5 | 19 |
| siVIM-269 | UGAACUCGGUGUUGAUGGCGU | 1 | 73 | 88 | 88 |
| siPLS3-1657 | AAUUAAAUCCACAACUGCCAA | 7 | 4 | 2 | 9 |
| siVIM-805 | UUGCUGACGUACGUCACGCAG | 7 | 83 | 89 | 89 |
| siVIM-812 | UUUCAUAUUGCUGACGUACGU | 0 | 0 | 22 | 5 |
| siMC4R-490 | UAUGAUGAUCCCAACCCGCUU | 6.8 | 86.3 | 90.7 | 90.2 |
| siLuc-1430 | CGGCGGCGGGAAGUUCACCGG | 20 | 66 | 79 | 80 |
| siCCNC-571 | UAUCAUGAAAGGAGGAUACAG | 14.5 | 45.6 | 74.3 | 77.8 |
| siLuc-309 | UUAUAAAUGUCGUUCGCGGGC | 8 | 15 | 27 | 24 |
| siOct-797 | UACAGAACCAUACUCGAACCA | 9 | 30 | 47 | 71 |
| siLuc-1120 | UUUCCCGGUAUCCAGAUCCAC | 17 | 11 | 41 | 56 |
| siCLTC-3114 | UUAAUAUACUCCAUAACACGU | 5 | 4 | 19 | 38 |
| siOct-670 | AAUGCUAGUUCGCUUUCUCUU | 13 | 74 | 90 | 87 |
| siLuc-49 | UCCAGCGGUUCCAUCUUCCAG | 2 | 82 | 92.6 | 93.9 |
| siGRK4-189 | AACUGCCUGAAGAGACGUCUU | 17 | 83 | 89 | 91 |
| siPLS3-1528 | ACCAUCUCCAAGAUCUUCCAG | 9 | 37 | 62 | 60 |
| siPLS3-1310 | UACUCCAGUCAACAGGAACUU | 2 | 31 | 43 | 57 |
| siKIF23-430 | UCGUUUAGCUUGAAAUGACCC | 0 | 25.4 | 47.9 | 58.8 |
| siCLTC-2416 | AAGUCGACUUGGAUUCACCUU | 10 | 66 | 85 | 87 |
| siLuc-1063 | CGCGCCCGGUUUAUCAUCCCC | 13 | 19.9 | 53 | 82.3 |
